# Treatment Options for Central Retinal Artery Occlusion

**DOI:** 10.1007/s11940-012-0202-9

**Published:** 2012-10-16

**Authors:** Sudha Cugati, Daniel D. Varma, Celia S. Chen, Andrew W. Lee

**Affiliations:** 1 Department of Ophthalmology, University of Adelaide, Adelaide, SA 5000 Australia; 2 Flinders Comprehensive Stroke Centre, Flinders Medical Centre, Bedford Park, SA 5042 Australia; 3 Department of Ophthalmology, Flinders Medical Centre and Flinders University, Flinders Drive, Bedford Park, SA 5042 Australia; 4 Flinders Comprehensive Stroke Centre, Flinders Medical Centre, Bedford Park, SA 5042 Australia

**Keywords:** Central retinal artery occlusion, Treatment, Thrombolysis, Thrombolytic therapy, Vasodilators, Intraocular pressure, Neovascularization

## Abstract

Central retinal artery occlusion (CRAO) is an ocular emergency and is the ocular analogue of cerebral stroke. It results in profound, usually monocular vision loss, and is associated with significant functional morbidity. The risk factors for CRAO are the same atherosclerotic risk factors as for stroke and heart disease. As such, individuals with CRAO may be at risk of ischemic end organ damage such as a cerebral stroke. Therefore, the management of CRAO is not only to restore vision, but at the same time to manage risk factors that may lead to other vascular conditions. There are a number of therapies that has been used in the treatment of CRAO in the past. These include carbogen inhalation, acetazolamide infusion, ocular massage and paracentesis, as well as various vasodilators such as intravenous glyceryl trinitrate. None of these “standard agents” have been shown to alter the natural history of disease definitively. There has been recent interest shown in the use of thrombolytic therapy, delivered either intravenously or intra-arterially by direct catheterisation of the ophthalmic artery. Whilst a number of observational series have shown that the recovery of vision can be quite dramatic, two recent randomised controlled trials have not demonstrated efficacy. On the contrary, intra-arterial delivery of thrombolytic may result in an increased risk of intracranial and systemic haemorrhage, while the intravenous use of tissue plasminogen activator (tPA) was not shown to be efficacious within 24 h of symptom onset. Nevertheless, both of these studies have shown one thing in common, and that is for treatment to be effective in CRAO, it must be deployed within a short time window, probably within 6 h of symptom onset. Therefore, while CRAO is a disease that does not have a treatment, nevertheless it needs to follow the same principles of treatment as any other vascular end organ ischaemic disease. That is, to attempt to reperfuse ischemic tissue as quickly as possible and to institute secondary prevention early.

## Introduction

Central retinal artery occlusion (CRAO) is the occlusion of the central retinal artery (CRA) with resultant infarction of the retina and vision loss. It was first described as an embolic occlusion of the CRA in a patient with endocarditis by von Graefe in 1859 [1]. The incidence of CRAO is estimated around 1.9/100,000 in the United States [2•].

Patients with CRAO typically present with an acute, painless loss of vision, and 80 % of affected patients have a final visual acuity of counting fingers or worse [3, 4]. Visual loss in CRAO occurs as a result of loss of blood supply to the inner retinal layers. Approximately 15–30 % of the general population have a cilioretinal artery, which is a branch of the short posterior ciliary artery [5]. It supplies a part or the whole of the fovea, and in those eyes where there is a CRAO, the cilioretinal artery is spared and the visual acuity may be preserved at 20/50 or better, with loss of peripheral vision only.

The most common cause of CRAO is thromboembolus, which occurs at the narrowest part of the central retinal artery, where it pierces the dural sheath of the optic nerve [6••, 7]. It could also occur as a result of an occlusive thrombus at the level immediately posterior to the lamina cribrosa [8]. Once the central retinal artery is occluded, the ability of the retina to recover depends on whether the offending embolus or thrombus is dislodged, and also on the retinal tolerance time [9, 10].

CRAO is divided into four distinct clinical entities:

1. Non-arteritic permanent CRAO (Fig. 1)

**Figure 1:**
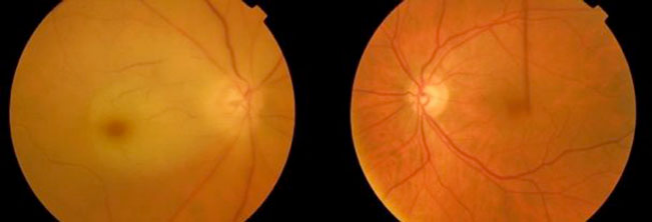
Colour fundus photograph showing non-arteritic permanent central retinal artery occlusion in the right eye and a normal fundus in the left eye.
  This group accounts for over two thirds of all CRAO cases, and is caused by platelet fibrin thrombi and emboli as a result of atherosclerotic disease [11–13].
2. Non-arteritic transient CRAO
  Non-arteritic transient CRAO (transient monocular blindness) accounts for 15 % of CRAOs and has the best visual prognosis. People suffering from this have a 1 % risk per year of having a permanent non-arteritic CRAO [14]. Transient vasospasm due to serotonin release from platelets on atherosclerotic plaques has also been suggested as a mechanism of transient CRAO in animal models [9].
3. Non-arteritic CRAO with cilioretinal sparing (Fig. 2)

**Figure 2:**
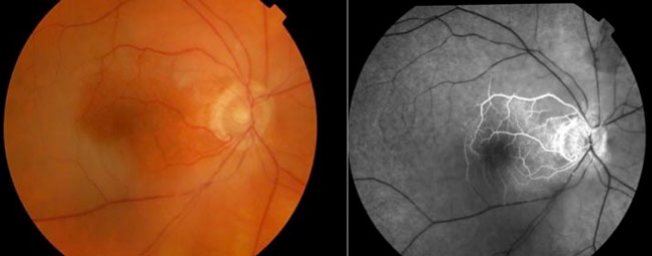
Colour fundus photograph and fundus fluorescein angiogram of the right eye showing non-arteritic CRAO with cilioretinal sparing.
  Preservation of the cilioretinal artery results in preserved perfusion of the macula region [4].
4. Arteritic CRAO (Fig. 3)

**Figure 3:**
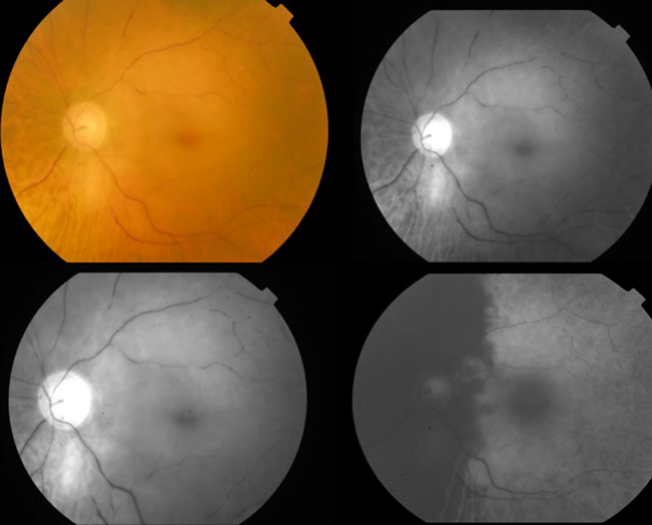
Colour fundus photograph of the left eye showing arteritic central retinal artery occlusion; serial fundus fluorescein angiogram showing delayed arterial filling and choroidal ischaemia.
  Arteritic CRAO (including the vasculitides) accounts for <5 % of cases. Giant cell arteritis is the most common entity in this category, and can cause bilateral visual loss. If an arteritic cause is suspected, it is essential to assess the inflammatory markers and treat urgently with systemic corticosteroids.

The majority of CRAOs are of the non-arteritic permanent type and are analogous to a terminal arterial branch occlusion in cerebral stroke. In a suspected non-arteritic CRAO due to thrombo-embolic cause, evaluation of atherosclerotic risk factors, including a family history of cerebrovascular and cardiovascular disease, diabetes mellitus, hyperlipidemia, a past history of vascular disease, smoking, palpitations, valvular heart disease or transient ischaemic events such as transient monocular blindness, transient ischemic attack (TIA) or angina symptoms, should be sought [15–22]. In those CRAOs where there are no atherosclerotic risk factors, especially in a young patient, other less common factors should be explored. These include the presence of vasculitis, sickle cell disease, myeloproliferative disorders, hypercoagulable states, and the use of the oral contraceptive pill or illicit drugs [23].

The following discussion on therapy concentrates mainly on the management of non-arteritic CRAO of presumed thromboembolic origin that forms the majority of CRAO cases.

## Treatment

### Diet & lifestyle

The risk factors for CRAO are those of atherosclerosis [24, 25]. Diet and lifestyle play a role indirectly for secondary prevention of further end organ ischemia. Hypertension and diabetes are the most frequent risk factors for CRAO [24]. Other associated risk factors include smoking, hypercholesterolemia and a family history of microvascular disease.

As a result, a diet with a low glycemic index would reduce the risk of vascular disease [26]; such a diet is rich in fruits, vegetables, grains, low-fat or nonfat dairy products, fish, legumes, poultry, and lean meats, polyunsaturated fatty acids. Also, regular exercise is essential to reduce the cardiovascular risk factors [27]. A healthy diet hence plays a role for secondary prevention in CRAO.

Hyperhomocystinemia is also recognised as a risk factor for CRAO [28], and results in dysfunction of vessel endothelium, with a proliferation of vascular smooth muscle and prothrombic hemostatic changes. Homocysteine levels are known to increase in a diet deficient in folic acid and vitamin B6 and B12, and hence diet with adequate folic acid and vitamin B6 and B12 in patients with hyperhomocystinemia would reduce the risk of CRAO.

### Pharmacological treatment

The retinal tolerance time to acute ischaemia has been evaluated in experimental studies [29]. These studies suggest that the retina in elderly, atherosclerotic and hypertensive rhesus monkeys suffer no detectable damage to CRAO for 97 min. Thereafter, partial recovery is possible if the ischemia is reversed up to 240 min. The time when irreversible retinal damage occurs with no recovery of vision is not known, but postulated to be around 6 to 6.5 h [30•, 31]. Hence, for any treatment to be effective, it is essential to implement the treatment within the correct time window. Spontaneous recanalization of the occluded central retinal artery may occur within 48 h to 72 h, but this may be partial [3]. Current reports on the visual improvement rate vary from 1 % to 10 % of cases of non-arteritic CRAO [13, 32]. Recent studies have suggested that the minimally invasive therapy in CRAO do not result in a significant visual recovery [33, 34].

Table 1 summarises the various available pharmacological options in the treatment of CRAO.

**Table 1:** Options available in treatment of central retinal artery occlusion

	Group of drugs	Mechanism of action
1.	Vasodilators	Increase the blood oxygen content
	Pentoxyphylline	
	Inhalation of carbogen	
	Hyperbaric oxygen	
	Sublingual isosorbide dinitrite	
2.	Ocular massage	Reduce intraocular pressure and hence increase the retinal artery perfusion or help dislodge the embolus
	Anterior chamber paracentesis	
	Intravenous acetazolamide	
	Intravenous mannitol	
	Topical antiglaucoma medications	
3.	Intravenous methylprednisolone	Reduction of retinal oedema
4.	Nd YAG Laser embolectomy	Lyse or dislodge the clot
5.	Intra arterial or intravenous thrombolysis	Help in thrombolysis of the embolus

#### Vasodilators, which help in increasing the blood oxygen content

##### Pentoxyphylline [35, 36]

Pentoxyphylline is a tri-substituted xanthine derivative that works by increasing erythrocyte flexibility, reducing blood viscosity, and increasing microcirculatory flow and tissue perfusion. It has been used in the treatment of peripheral vascular disease. In a randomised control trial, a small number of patients (ten patients) with CRAO were randomised to either pentoxiphylline or placebo for 4 weeks. The endpoint measurements include objective CRA blood flow measured by duplex scanning. The authors noted an increase in the peak systolic and end diastolic flow velocities by 550 % and 400 %, respectively, in five patients treated with pentoxifylline, versus 288 % and 200 %, respectively, in a placebo group of five patients. However, the visual recovery was not discussed in the study and the numbers were small [36].

Standard dosage: 600 mg tds
Contra indications: Allergy to theophylline or caffeine
Main drug interactions: Warfarin: Pentoxiphylline increases the anticoagulant effect of warfarin
  Theophylline & Aminophylline: When used in combination, pentoxiphylline potentiates the effect of theophylline and aminophylline
Main side effects: Digestive (dry mouth or dehydration, constipation, anorexia, cholecystitis)
  Neurogenic (aseptic meningitis, seizures, confusion, depression anxiety), Cardiovascular (hypotension, edema, dyspnea)
  Respiratory (nasal congestion, nosebleed, breathing difficulty)
  Dermatological (rash, angioedema, urticaria, pruritus, brittle fingernails)
  Ear and eye related (earache, scotoma, conjunctivitis, blurred vision)
Cost / cost effectiveness: Pentoxyphylline is inexpensive, but the effectiveness is not established.

##### Inhalation of carbogen (mixture of 95 % oxygen and 5 % carbon dioxide)

Carbogen is a mixture of 4–7 % carbon dioxide and 93–96 % oxygen, used in the treatment of CRAO based on the assumption that carbon dioxide will prevent oxygen-induced vasoconstriction and hence maintain or even improve the blood flow while maintaining the oxygenation of the retina [37, 38]. Carbogen inhalation is conducted for 10 min every hour during waking hours, and 10 min every 4 h at night and continued for 48–72 h. However, retinal dynamics obtained with carbogen have been contradictory [39], and the results of visual recovery with carbogen in acute CRAO was not significant [40].

##### Hyperbaric oxygen

The proposed role for hyperbaric oxygen in CRAO is an increase the partial pressure of oxygen delivery to ischemic tissue until spontaneous or assisted reperfusion occurs. The exact pathogenesis is debated [41, 42, 43•, 44–46] and the efficacy is not proven. The protocol for hyperbaric oxygen varies in different studies, with an average of 2–2.5 atm for approximately 90 min within 8 h of onset of CRAO [47].

##### Sublingual isosorbide dinitrite

Nitroglycerin causes relaxation of vascular smooth muscle by stimulating intracellular cyclic guanosine monophosphate (GMP). Nitroglycerine has been used in central retinal artery occlusion along with other modes of treatment, including ocular massage and other means to reduce the intraocular pressure [48].

Standard dosage: 10 mg
Contraindications: Hypersensitivity, severe anaemia, recent use of phosphodiesterase inhibitors
Main drug interactions: Drugs which decrease the effect of isosorbide dinitrite include:
  - Dopamine receptor agonist (Bromocriptine, cabergoline)
  - Ergopeptides (dihydroergotamine, ergotamine, methylergonovine, methysergide)
  - Drugs which have an additive vasodilator effect
  - Phosphodiesterase inhibitors
Main side effects: Common: Headache, hypotension, tachycardia, dizziness, lightheadedness, blurred vision, flushing, nausea and vomiting, nervousness, xerostomia
  Serious: Methemoglobinemia (rare), syncope, prolonged bleeding time, exfoliative dermatitis, unstable angina, rebound hypertension, thrombocytopenia
Cost / cost effectiveness: Isosorbide dinitrate is inexpensive, but the effectiveness is not established.

#### Reduction of intraocular pressure, and hence increase the retinal artery perfusion or help dislodge the embolus

As mean ocular perfusion pressure is the difference between mean arterial pressure and intraocular pressure, attempts have been made to reduce the intraocular pressure and thus increase ocular perfusion [33, 48].

##### Ocular massage

Ocular massage includes the compression of the globe with a three-mirror contact lens for 10 s, to obtain retinal artery pulsation or flow cessation if the pulsation is not seen followed by a 5 s release [16, 48, 49]. Alternatively, digital massage can be applied over the globe over the closed eyelids for 15–20 min. The combination of the massage and acetazolamide can decrease the intraocular pressure to as low as 5 mmHg within a short period of time [49]. Ocular massage causes retinal arterial dilatation and large fluctuations in Intraocular pressure (IOP). It has been postulated that this activity may mechanically facilitate the disintegration of a thrombus, or dislodge an impacted embolus into a more peripheral part of the retinal circulation [18].

##### Intravenous acetazolamide

Acetazolamide is a carbonic anhydrase inhibitor that reduces the aqueous production and hence reduces the intraocular pressure. This in turn increases the retinal perfusion [48].

Standard dosage: intravenous injection 500 stat or 250 mg qid for 24 h
Contraindications: Hypokalemia & hyponatremia, hyperchloremic acidosis, sulfa allergy, liver or renal disease including liver cirrhosis
Main drug interactions: Methenamine: When methenamine is used with acetazolamide, there are insoluble precipitates in the urine, and this decreases the effect of both drugs
  Cisapride: Acetazolamide increases the toxicity of cisapride by passive tubular resorption by increasing the pH
Main side effects: CNS: Confusion, Convulsions, Drowsiness, Flaccid paralysis, Malaise
  GI: Anorexia, Diarrhea, Metallic taste, Nausea, Vomiting, Hepatic disease, Melena
  Blood: Aplastic anemia, Agranulocytosis, Leukopenia Paresthesia, Thrombocytopenia, Thrombocytopenic purpura
  Renal: Hematuria, Polyuria,
  Electrolyte imbalance, Glycosuria, acidosis
  Photosensitivity, Urticaria
  Hearing dysfunction or tinnitus
  Sulfonamide type reaction
Special points: The medication is used as an immediate intravenous or short-term intensive oral therapy but not designed as a long-term therapy. Hence, the long-term monitoring in acetazolamide use do not apply in this setting.
Cost / cost effectiveness: Acetazolamide is inexpensive.

##### Intravenous mannitol

Standard dosage: 1.5–2 g/ Kg over 30–60 min
Contraindications: Hypersensitivity, severe dehydration, anuria, progressive renal disease, severe pulmonary edema or heart failure, metabolic edema, active intracranial bleeding
Main drug interactions: Tobramycin: Mannitol increases the level of tobramycin by unknown mechanism
  Lurasidone, Nitroglycerine: these can increase the effect of mannitol
Main side effects: GI: Nausea, Vomiting
  CVS: Angina-like chest pains, hypotension, congestive cardiac failure, Phlebitis
  CNS: Convulsions, Dizziness,
  Electrolyte imbalances, acidosis
  Blurred vision
  Renal: Urinary retention
Cost / cost effectiveness: Mannitol is inexpensive.

##### Topical antiglaucoma drops

Glaucoma medications aim to lower the intraocular pressure and increase the gradient across the optic nerve head (perfusion pressure = mean arterial pressure – intraocular pressure). The drops act by decreasing aqueous humor production (with beta-adrenergic blockers or carbonic anhydrase inhibitors), or increasing outflow (with prostaglandin analogue or an alpha-agonist). However, the onset of action is slow with topical drops compared to intravenous acetazolamide and mannitol.

#### Reduction of retinal oedema

##### Intravenous methylprednisolone

A single dose of intravenous methylprednisolone of 1 g has been reported in a case series of patients whose vision failed to improve following the conventional treatment of measures to reduce the intraocular pressure [50, 51]. The possible mechanism of action is reported to be a reduction in the retinal oedema, and hence, improvement of vision. However, the patients were younger, and it is possible that these patients had vasospasm or CRAO secondary to vasculitis, rather than atherosclerotic-induced CRAO.

Special consideration: Intravenous methylprednisolone is given in suspected arteritic CRAO due to giant cell arteritis, but is not a recommended treatment for non-arteritic permanent CRAO.

#### Thrombolytic therapy

##### Tissue plasminogen activator (tPA)

Tissue plasminogen activator (tPA) is a naturally occurring fibrinolytic agent found in vascular endothelial cells and results in clot lysis. At the site of the thrombus, the binding of tPA and plasminogen to the fibrin surface induces a conformational change that facilitates the conversion of plasminogen to plasmin and dissolves the clot.

Alteplase, a recombinant tPA, is a fibrin- specific agent commonly used in ischaemic stroke, myocardial infarction and massive pulmonary embolism. CRAO is a stroke of the eye analogous to ischemic cerebral stroke, and the CRA and retina are homologous to the circle of Willis and the brain respectively. Fibrinolysis is an accepted standard therapy in the treatment of ischaemic stroke, and hence there is biological plausibility for its use in CRAO.

Two large reviews [52, 53] and several observational studies [31, 54, 55] have suggested that thrombolysis in the treatment of CRAO may improve the visual acuity with few serious complications. However, data from a large multicentre randomised study using intra-arterial thrombolysis [56••], and another randomised study using intravenous thrombolysis [30•] have failed to show an improvement in the visual acuity.

Intravenous thrombolytic delivery has the advantage of easier access without the need for specialized interventional radiology unit and reduced risk of complications [57]. On the other hand, the disadvantage of an endovascular intra-arterial approach is the increased risk of strokes, the requirement of a neuro-interventionalist, and a longer procedural time [58]. Not only are the results in various studies debatable, there is a marked heterogeneity of drug regimens, pre-treatment and post-treatment evaluation, and the therapeutic window (time from the onset of visual loss to drug infusion), which make it very difficult to draw conclusions from the published studies [59].

Specific drugs: Various thrombolytic agents have been indicated in different studies. Most often, tPA or urokinase are used.
Standard dosage: Intravenous tPA dose varied from a loading dose of 50 mg infusion over 60 min (a dose smaller than recommended in ischaemic stroke) to 0.9 mg/ Kg (maximum of 90 mg) that is the standard dose used in ischemic stroke [59]. The use of adjuvant therapy, such as heparin with intravenous heparin, or anti-platelet therapy is not standardized, and was greatly varied between the various studies to allow comparison and comment.
Contraindications []
  - Evidence of intracerebral hemorrhage on pre-treatment evaluation
  - Suspicion of subarachnoid hemorrhage on pretreatment evaluation
  - Myocardial infarction, Intracranial or intraspinal surgery, serious head trauma or stroke in the last 3 months
  - Gastro-intestinal or genito-urinary haemorrhage in the previous 3 weeks
  - Early ischaemic changes occupying greater than one-third of the middle cerebral artery (MCA) territory
  - History of intracerebral hemorrhage (ICH)
  - Uncontrolled hypertension at time of treatment (>185 mmHg systolic or >110 mmHg diastolic)
  - Blood glucose >22.22 mmol [62]
  - NIH Stroke scale score >25 [62]
  - Seizure at onset of stroke
  - Active internal bleeding
  - Intracranial neoplasm, arteriovenous malformation, or aneurysm
  - Known bleeding diathesis, including but not limited to :
  - Current use of oral anticoagulants such as Warfarin or an International normalised ratio (INR) of >1.7, prothrombin time (PT) of >15 s
  - Administration of heparin within 48 h preceding the onset of the stroke and a elevated activated partial thromboplastin time (aPTT) at presentation
  - Platelet count <100,000 mm^2^
Main drug interactions []: Increased risk of bleeding with the concomitant or immediate prior use of anti-coagulant, anti-platelet or vitamin K antagonists.
  No studies on interactions of tPA and other cerebroactive drugs.
Main side effects []: Intracerebral and systemic haemorrhage
  The following have been reported in clinical trials and post marketing experience involving tPA: anaphylactoid reaction, laryngeal edema, orolingual angioedema (often due to concomitant use of ace-inhibitors), rash and urticaria.
  Specific to ischaemic stroke: cerebral edema, cerebral herniation, seizure, new ischaemic stroke.
Special considerations: The role of adjuvant therapy with thrombolysis is not known. There is no consensus with regard to the standard dose, route of administration or simultaneous use of heparin in various studies. The EAGLE study used intra-arterial tPA within 24 h of visual loss followed by heparin for 5 days [60].
Cost / cost effectiveness: The agent tPA cost approximately USD $1,487.06 per 50 mg vial and $2,974.13 per 100 mg vial (including diluent) [63]. It requires specialized neuro-intervention set-up and neuroradiologist for intra-arterial administration, or specialized stroke unit set-up for intra-arterial administration.
  Cost effectiveness has not been established in CRAO. However, a recent systematic review in The American Journal of Managed Care on cerebral ischaemic stroke has built upon primary cost effectiveness data from Fagan and colleagues in 1998 [64, 65]. The study used data from the NINDS rt-PA Study [66] and medical literature to estimate health and economic outcomes in ischaemic stroke patients treated with rt-PA. A Markov model was then developed to estimate costs per 1,000 patients eligible for rt-PA vs. costs per 1,000 people untreated [64]. The estimated impact of rt-PA use on long term health outcomes was a savings of 564 quality-adjusted life years (QALYs) over 30 years in the 1,000 person model [64].
  Cost effective calculations showed rt-PA treatment resulted in incremental cost savings of $8,000 per QALY gained. Using Fagan’s cost saving result at $600 per rt-PA treated, it was estimated that in 2005, for every 2 % increase in the proportion of ischaemic stroke patients receiving rt-PA, the result was $7 million dollars of annual healthcare cost savings in the USA [67].

### Surgery/procedures

#### Lowering intraocular pressure to increase optic nerve head perfusion gradient

##### Anterior chamber paracentesis

This is achieved by inserting a 27-gauge needle into the anterior chamber via the limbus and withdrawing 0.1 ml to 0.2 ml of aqueous fluid. The potential benefits of paracentesis include a dramatic drop in IOP, and dilatation of the retinal arteries because of the vascular tortuosity resulting from distortion of the globe [49]. However, a maximum increase in retinal arterial volume flow of only 20 % has been estimated from animal studies, and a rise in perfusion pressure of less than 15 % is expected when the IOP falls from 15 mmHg to 5 mmHg [18, 40].

Complication: The potential risk of paracentesis includes infection.
Special points: The procedure is well described and commonly employed [62], but the effectiveness is not proven.
Cost: The cost of the procedure is inexpensive.

#### Means to lyse and dislodge the embolus

##### Nd YAG laser (Neodymium:yttrium-aluminum-garnet laser)

A single case report has been reported with an improvement of vision following the use of Nd YAG laser in a patient with CRAO. The laser, using 0.8–1.1 mJ intensity, was focused slightly posterior to the visible arterial wall at the site of the embolus [68, 69]. The laser tends to dislodge the embolus, which dissolves later. The complication of the laser includes vitreous hemorrhage and false aneurysm. Since these are only case reports and the procedure involves significant complications, YAG laser embolectomy is not accepted as a standard treatment of choice in CRAO.

##### Pars plana vitrectomy

A pars plana vitrectomy followed by removal of the embolus has been shown to be promising in case reports [70, 71] and small case series [72]. It has been suggested to perform a fluorescein angiogram to ascertain the site of occlusion, and a longitudinal incision is made over the area of occlusion to remove the embolus. However, the studies are limited and randomised control studies are required to ensure the safety of the treatment of this procedure in CRAO.

Complications: Vitreous haemorrhage
  Cataract
Special points: This procedure is not a standard acute treatment for CRAO.
Cost/cost effectiveness: Expensive when considering the cost of surgery and expertise required. The treatment is not proven, and hence not cost effective.

### Neovascularisaion following CRAO

#### Treatment of neovascularization

**Figure 4:**
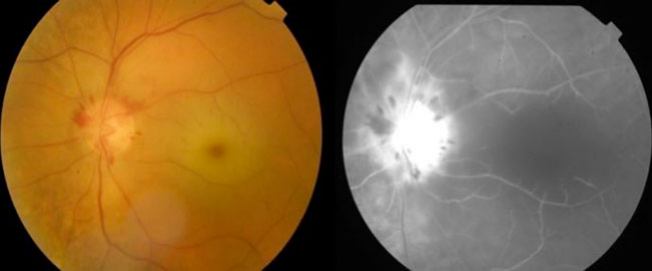
Colour fundus photograph and fundus fluorescein angiogram of the left eye showing neovascularisation of the disc where the new vessel result in leakage and bright hyper-fluorescence at the disc.

Neovascularization (Fig. 4) followed by glaucoma occurs in 2.5–31.6 %, and the mean time from CRAO to observed neovascularization was 8.5 weeks (range 2–16 weeks) [73]. Rudkin et al. reported a definite temporal relationship between the CRAO and neovascularization events in 18.2 % of patients, with no other causes of neovascularization demonstrable in their cohort of patients. In the majority of cases of neovascularisation, there were no clinical features of ocular ischemia, and no association with a hemodynamically significant stenosis of the carotid artery. It is essential to review all patients with acute CRAO at regular intervals as early as 2 weeks, then monthly up to 4 months, post-CRAO. If at any stage, a patient develops neovascularisation, panretinal photocoagulation should be performed promptly. Pan-retinal photocoagulation aims to decrease demand for oxygen in the peripheral retina to reduce the vascular endothelial vascular growth factors that cause abnormal blood vessel. The new vessels, if left untreated, can bleed easily into the eye and result in vitreous hemorrhage or high pressure in the eye (neovascular glaucoma).

## Conclusion

CRAO should be considered as an ocular emergency and is the ocular analogue of cerebral stroke. The same atherosclerotic risk factors that predispose to cardio, peripheral and cerebrovascular diseases are present in CRAO, and these must be actively evaluated to prevent further medical comorbidities. Current acute managements in acute CRAO have limited efficacy to improve vision and studies suggest that for treatment to be effective in CRAO, it must be deployed within a short time window, potentially within 6 h of symptom onset within the retinal tolerance time for any therapy to be effective. Current management follows the same principles of treatment as any other vascular end organ ischaemic disease, and includes vascular review to prevent further end organ ischemia and ocular complications.
